# Morpholino-Mediated Isoform Modulation of Vascular Endothelial Growth Factor Receptor-2 (VEGFR2) Reduces Colon Cancer Xenograft Growth

**DOI:** 10.3390/cancers6042330

**Published:** 2014-11-26

**Authors:** Brian C. Stagg, Hironori Uehara, Nathan Lambert, Ruju Rai, Isha Gupta, Bryce Radmall, Taylor Bates, Balamurali K. Ambati

**Affiliations:** John A Moran Eye Center, University of Utah, Salt Lake City, UT, 65 Mario Capecchi Drive, Salt Lake City, UT 84132, USA; E-Mails: hironori.uehara@hsc.utah.edu (H.U.); nathan.lambert@hsc.utah.edu (N.L.); rurai@bu.edu (R.R.); isha.gupta@hsc.utah.edu (I.G.); radmall@ohsu.edu (B.R.); tbates.j@gmail.com (T.B.); bambati@gmail.com (B.K.A.)

**Keywords:** VEGFR2, angiogenesis inhibitors, morpholinos, alternative polyadenylation

## Abstract

Angiogenesis plays a key role in tumor growth. Vascular endothelial growth factor (VEGF) is a pro-angiogenic that is involved in tumor angiogenesis. When VEGF binds to membrane-bound vascular endothelial growth factor receptor 2 (mVEGFR2), it promotes angiogenesis. Through alternative polyadenylation, VEGFR2 is also expressed in a soluble form (sVEGFR2). sVEGFR2 sequesters VEGF and is therefore anti-angiogenic. The aim of this study was to show that treatment with a previously developed and reported antisense morpholino oligomer that shifts expression from mVEGFR2 to sVEGFR2 would lead to reduced tumor vascularization and growth in a murine colon cancer xenograft model. Xenografts were generated by implanting human HCT-116 colon cancer cells into the flanks of NMRI nu/nu mice. Treatment with the therapeutic morpholino reduced both tumor growth and tumor vascularization. Because the HCT-116 cells used for the experiments did not express VEGFR2 and because the treatment morpholino targeted mouse rather than human VEGFR2, it is likely that treatment morpholino was acting on the mouse endothelial cells rather than directly on the tumor cells.

## 1. Introduction

Angiogenesis, or the growth of new blood vessels, plays a key role in tumor growth and invasion [1,2]. Because of this, the reduction of tumor angiogenesis is an important target in cancer treatment. Multiple factors influencing blood vessel growth have been identified, including vascular endothelial growth factor (VEGF), platelet derived growth factor (PDGF), fibroblast growth factor (FGF), neuropilin (NRP), and cadherin [3,4,5,6,7]. VEGF has been shown to play a particularly important role in tumor angiogenesis and is the target of several anti-cancer medications [8,9]. Targeting VEGF has been shown to be useful in treatment of several cancers, including colon cancer, ovarian cancer, and glioblastoma multiforme [10,11,12].

VEGF binds to several different receptors [13]. The primary angiogenic receptor for VEGF is vascular endothelial growth factor receptor 2 (VEGFR2), which is also referred to as KDR [14,15]. Because of alternative polyadenylation, VEGFR2 is expressed in two different isoforms, membrane-bound VEGFR2 (mVEGFR2) and soluble VEGFR2 (sVEGFR2) [16,17]. mVEGFR2 is pro-angiogenic and consists of 7 extracellular domains, a transmembrane domain, and tyrosine kinase domains [14,15]. sVEGFR2 is composed of only the extracellular portion of the molecule and does not contain the tyrosine kinase domains; therefore sVEGFR2 is anti-angiogenic and anti-lymphangiogenic [16,18].

Anti-sense technology has been used in the past to induce alternative splicing [19,20]. Our laboratory has previously developed and reported an antisense morpholino oligomer directed against the exon 13-intron 13 junction that shifts expression from mVEGFR2 to sVEGFR2 [18]. We previously showed that this morpholino reduces mVEGFR2 and increases sVEGFR2 at both an mRNA and protein level. Using this morpholino, we successfully reduced laser photocoagulation induced choroidal neovascularization with intravitreal injection, decreased corneal neovascularization with subconjunctival injection, and suppressed corneal graft rejection with subconjunctival injection [18]. As our morpholino was successful in treating ocular models of neovascularization, we hypothesized that the morpholino would successfully reduce tumor neovascularization and growth.

## 2. Results and Discussion

### 2.1. In Vitro HCT 116 Cells Do Not Express VEGFR2 in Either Isoform

**Figure 1:**
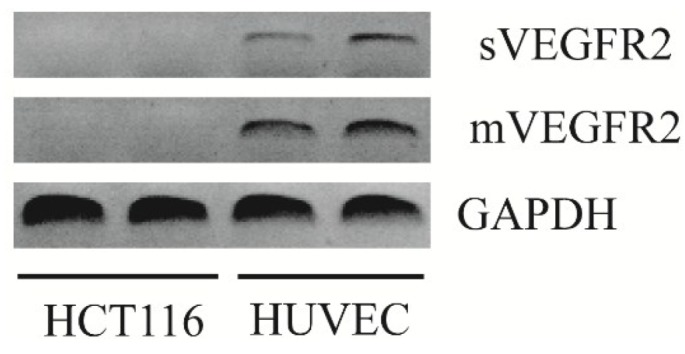
PCR was used to evaluate *in vitro* HCT 116 cells for VEGFR2 expression. This 1.2% agarose electrophoresis image shows no expression of mVEGFR2 and sVEGFR2 by HCT 116 cells, but positive expression of both by HUVEC controls.

**Figure 2:**
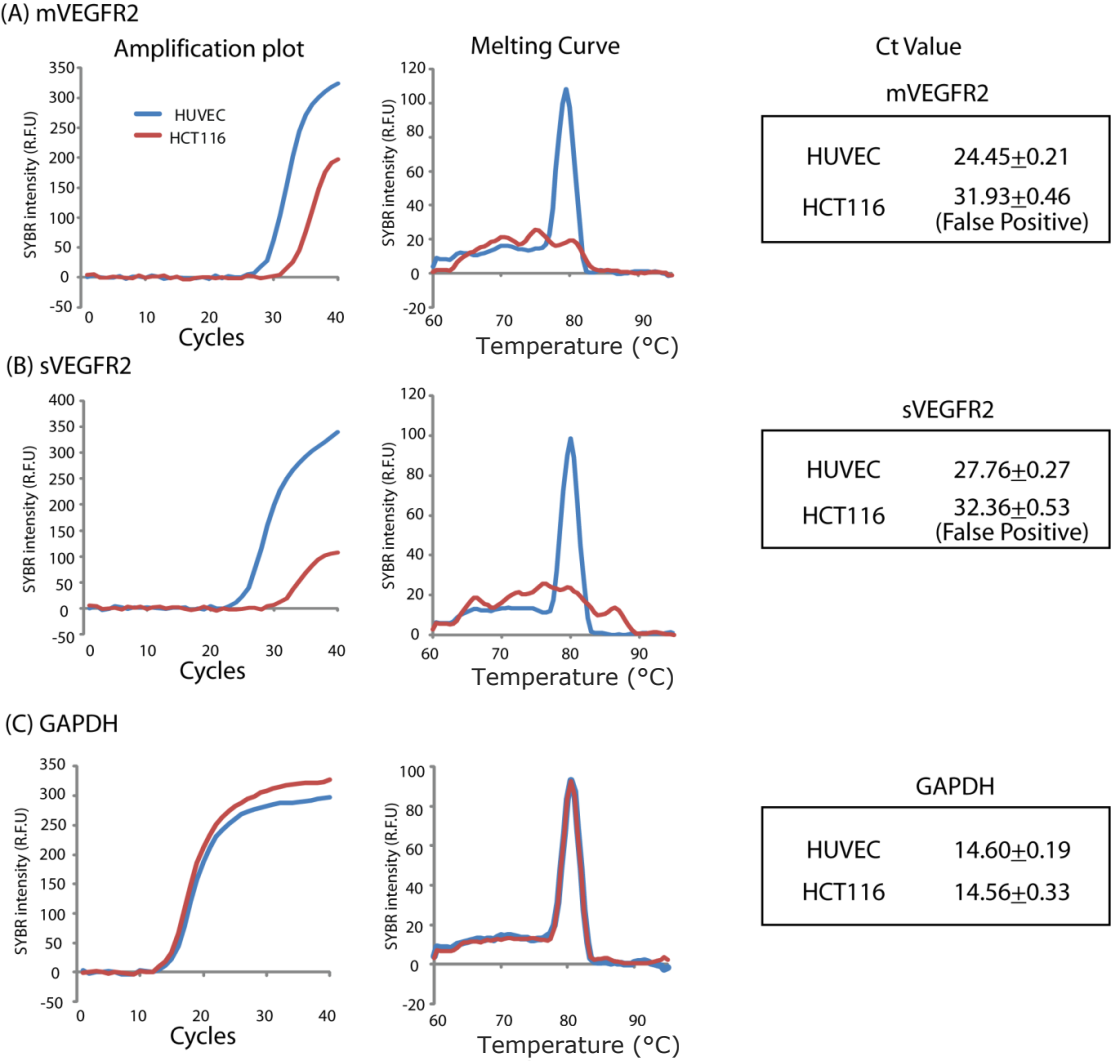
Real-time PCR also showed that the *in vitro* HCT 116 cells did not express VEGFR2 in either isoform. This confirmed the RT-PCR results presented in Figure 1. In this figure, the HCT116 does show some elevation, however the melting curve does not show a peak so this is a false positive result.

To ensure that the treatment morpholino was acting only on the effects of VEGFR2 on the vasculature, we used a xenograft model and verified that the human cancer cells that we used (HCT116) did not express VEGR2. RT-PCR showed that *in vitro* HCT 116 cells did not express VEGFR2 in either of its isoforms, while HUVEC control did (Figure 1). This result was confirmed with real-time PCR (Figure 2).

### 2.2. sVEGFR2-Inducing Morpholino Decreases Proliferation of Endothelial Cells but Does Not Decrease Proliferation of HCT116 Cells in Vitro

**Figure 3:**
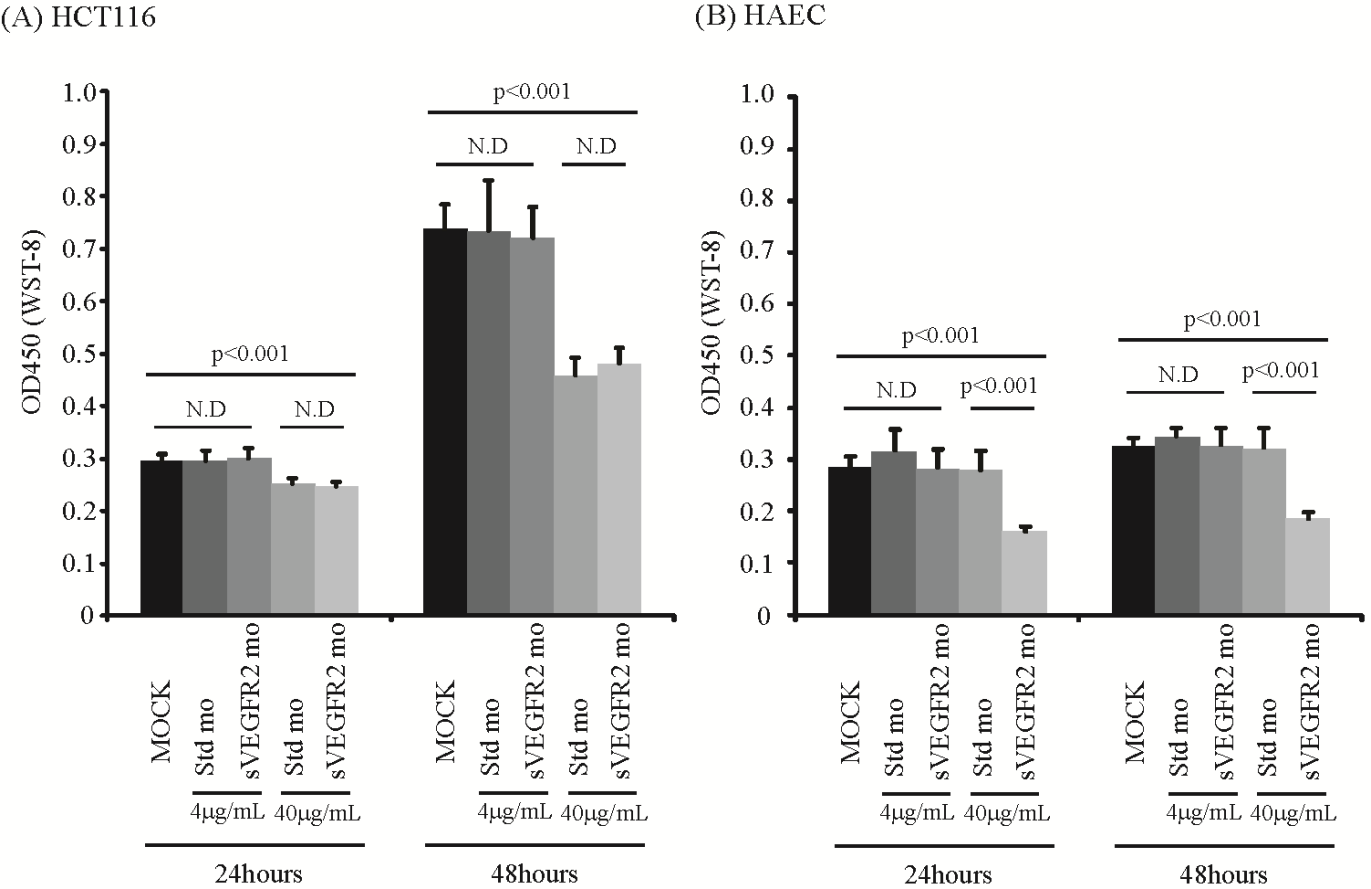
At concentrations of 4 μg/mL, the control and treatment morpholino did not affect neither HAEC proliferation nor HCT 116 proliferation at both 24 and 48 h. At higher concentrations of 40 μg/mL, the treatment morpholino decreased proliferation of HAEC but the control morpholino did not. At the higher concentration, both the control and treatment morpholinos decreased HCT116 proliferation (*p* < 0.001).

To confirm that the sVEGFR2-inducing morpholino decreases endothelial cell proliferation but does not have direct effect on HCT116 cells, *in vitro* cell proliferation assays were performed. At a lower concentration of 4 micrograms/milliliter, neither the standard morpholino nor the sVEGFR2-inducing morpholino affected proliferation of both Human aortic endothelial cells (HAEC) and HCT116. At a higher concentration of 40 micrograms/milliliter, the sVEGFR-2 inducing morpholino decreased proliferation of HAEC but the standard morpholino did not. However, at the higher concentration, both the standard morpholino and the sVEGFR2-inducing morpholino decreased HCT116 proliferation (*p* < 0.001) (Figure 3). As both the control and treatment morpholino exhibited this effect, it is most likely related to toxicity of the morpholino at a high concentration on the more fragile HCT 116 cells rather than changes of VEGFR2 expression. This toxicity is possible due to dendrimer formation [21]. These results suggest that the sVEGFR2-inductin morpholino has a direct effect on the endothelial cells but no direct effect on the HCT-116 cells.

### 2.3. Treatment with sVEGFR2-Inducing Morpholino Decreases Tumor Growth in Vivo

**Figure 4:**
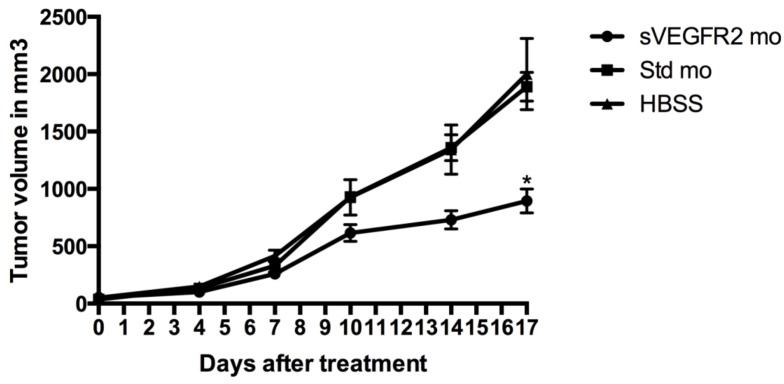
The graph shows tumor volume at 4, 7, 10, 14, and 17 days after initiation of treatment. There was a statistically significant difference between the sVEGFR2-inducing morpholino group and both controls (* denotes *p* = 0.035) at 17 days after initiation of treatment, but no statistically significant difference between the controls.

HCT116 cells were injected subcutaneously into the flank of 6-week-old NMRI nu/nu mice (Jackson Laboratories, Farmington, CT, USA). One week after injection of the cells, treatment was initiated with the sVEGFR2-inducing morpholino, a standard control morpholino, or HBSS as a control. Seventeen days after initiation of treatment, the average tumor volumes were 895 mm^3^ in the sVEGFR2-inducing morpholino group, 1,890 mm^3^ in the standard morpholino group, and 1,935 mm^3^ in the HBSS group (*n* = 5) (Figure 4). There was a statistically significant difference between the sVEGFR2-inducing morpholino group and both controls (*p* = 0.035) at 17 days after initiation of treatment, but no statistically significant difference between the controls.

### 2.4. Treatment with sVEGFR2-Inducing Morpholino Decreases Tumor Vascularization

**Figure 5:**
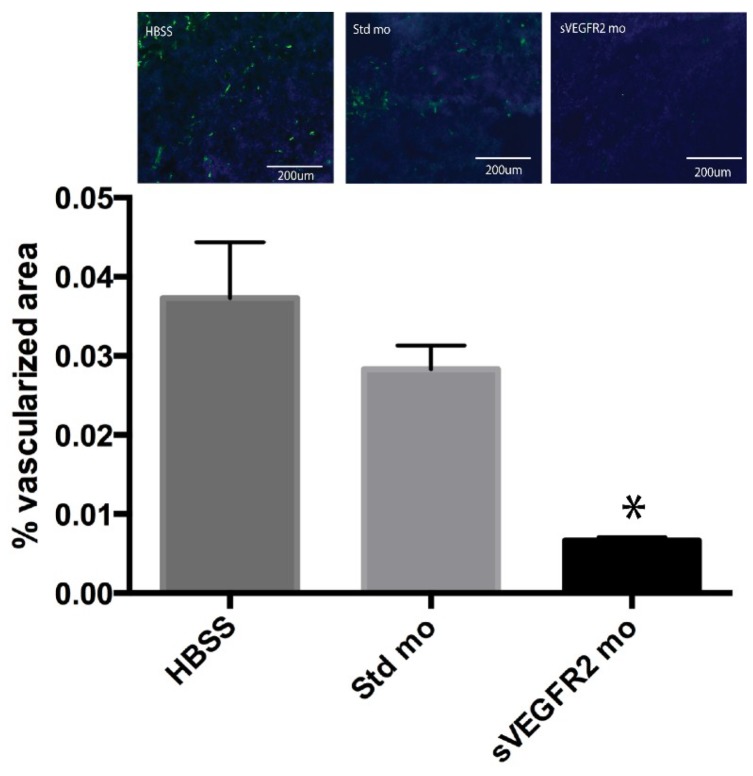
The graph shows average percentage of vascularized area of histological sections of tumors from each of the experimental groups. There was a statistically significant difference between the sVEGFR2-inducing morpholino group and both controls, but no statistically significant difference between the controls (* denotes statistical significance, *p* = 0.03 for sVEGFR2 to Std and *p* = 0.05 for sVEGFR2 to HBSS). Above the graph is a representative image from the HBSS, standard morpholino (Std mo), and sVEGFR2-inducing morpholino (sVEGFR2 mo) groups. Blue is DAPI staining and green is Isolectin staining for blood vessels.

Seventeen days after the initiation of treatment, tumors were harvested for vascularization analysis. Fluorescent microscopy of tumor sections using staining for endothelial cells showed that the percentage of tumor area covered by blood vessels was 0.67% in the sVEGFR2-inducing morpholino group, 2.8% in the standard morpholino group, and 3.7% in the HBSS group (*n* = 4). There was a statistically significant difference between the sVEGFR2-inducing morpholino group and both controls, but no statistically significant difference between the controls (Figure 5, *p* = 0.03 for sVEGFR2 to Std and *p* = 0.05 for sVEGFR2 to HBSS).

## 3. Experimental Section

### 3.1. Morpholino Oligomers

Morpholino oligomers were purchased from Gene Tools (Philomath, OR, USA). The sequence of the treatment morpholino is 5'-CACCCAGGGATGCCTCCATACCTAG-3'. We have previously shown that this morpholino shifts expression of from mVEGFR2 to sVEGFR2 in mice at both a protein and an mRNA level [18]. Standard morpholino oligomers were used as a control. The sequence for the standard nonsense morpholinos is 5'-CCTCTTACCTCAGTTACAATTTATA-3'.

### 3.2. Tumor Cells

HCT 116 (human colon cancer) cells were used for all experiments (American Type Culture Collection (ATCC, Manassas, VA, USA) following the manufacture’s protocols. PCR was used to evaluate HCT 116 cells for VEGFR2 expression. After culturing for three days in a six well plate, total RNA was extracted by RNAeasy kit (Qiagen, Valencia, CA, USA). After DNaseI treatment (Sigma-Aldrich, St. Louis, MO, USA), 750 ng total RNA was used for reverse transcription (Omniscript, Qiagen) using Oligo dT. An aliquot of cDNA (75 ng, 2 mL) was used for PCR. The sVEGFR2 primer sequence (forward 5'-ACCAAGGCGACTATGTTTGC-3', reverse 5'-CAATTCTGTCACCCAGGGAT-3') and the mVEGFR2 primer sequence (forward 5'-ACCATTGAAGTGACTTGCCC-3', reverse 5'-CCGGTTCCCATCTCTCAGTA-3') have previously been reported [18]. The PCR conditions were 95 °C for 2 min, then 30 cycles of 94 °C for 15 s, 55 °C for 30 s and 72 °C for 30 s. Samples were also run for 40 cycles. Samples were run in 1.2% agarose electrophoresis. The PCR did not show any expression of either mVEGFR2 or sVEGFR2 by the HCT116 cells after either 30 cycles or 40 cycles. To confirm this result, real-time PCR was performed. For real-time PCR, 1 mL (37.5 ng) of cDNA was used for SYBR green real-time PCR (Qiagen) in 25 mL with 0.3 µM primers. The CFX96 real-time PCR detection system was used (BioRad, Hercules, CA, USA). The real-time PCR conditions were 95 °C for 15 min, 94 °C for 15 s, then 40 cycles of 55 °C for 30 s and 72 °C for 30 s. After the reaction, the melting curve was from 60 °C to 95C. N = 4, Ct value is reported as average ± standard deviation. Statistics were not performed because HCT116 showed a false positive. Absence of HCT116 expression of VEGFR2 in either isoform was confirmed to ensure that any effect of the treatment morpholino would be as a result of acting on the murine neovascularization and not a direct effect on the tumor cells. 

### 3.3. Cell Proliferation Assay

The cell proliferation assay was performed using Cell Counting Kit-8 (Dojinod, Rockville, MD, USA) following the manufacture’s protocol. Briefly, 1,000 cells of either human aortic endothelial cells (HAEC, Lonza, Basel, Switzerland) or HCT116 were plated to a 96 well plate with 100 mL of 10% FBS/McCoy5'A. The next day, 10 mL of standard morpholino, sVEGFR2-inducing morpholino, or mock was added. We tested 4 μg/mL and 40 μg/mL concentrations of mopholinos. After 24 and 48 h, 10 mL of cell counting-8 kit solution was added to each well. After 1 h of incubation, the absorbance of 450 nm was measured.

### 3.4. Mice and Tumor Xenograft Model

All mice-related procedures were reviewed and approved by the University of Utah Institutional Animcal Care and Use Committee (protocol number 11-03008, approved March 7, 2011) and are in accordance with the Animal Research: Reporting of *In Vivo* Experiments (ARRIVE) guidelines. Care was taken to ensure that the minimum number of animals was used and that harm to the animals was minimized. 6-week-old NMRI nu/nu mice (Jackson Laboratories, Farmington, CT, USA) were used for xenograft studies. The xenograft studies were conducted using methods previously described [22,23]. Briefly, HCT116 cells were prepared and cared for using the standard manufacturer’s protocol. 3.0 × 10^6^ cells were injected subcutaneously into the flank of each mouse. One week after injection of the cells, treatment was initiated. The treatment group received the sVEGFR2-inducing morpholino (500 ng/mL, 50 mL injection) and the control groups received either standard morpholino 500 ng/mL, 50 mL) or HBSS (50 mL injection). Tumors were directly injected with treatment twice weekly for a period of 17 days. Direct tumor injection was used rather than systemic injection to limit the amount of morpholino required. Tumor size was measured using calipers twice weekly and tumor volume was estimated using the formula volume = (width)^2^ × length/2. The volumes at days 4, 7, 10, 14, and 17 were averaged and a one-tailed Student’s t-test was used to evaluate for statistical significance at each time point (*n* = 5).

### 3.5 Tumor Vascularization Analysis

Seventeen days after the initiation of treatment, tumors were harvested for vascularization analysis. To evaluate the effect of treatment on tumor vascularization, fluorescent staining was performed on tumor sections. After tumors were harvested, they were fixed in 4% PFA. Each cryosection was stained by Isolectin conjugated Alexa488 (Invitrogen, #I21411, Grand Island, NY, USA) for blood vessel staining. Isolectin GS-IB4 has been used previously for endothelial staining and some studies have shown its binding of endothelial cells [24,25,26,27]. Images of the slides were captured by confocal microscope (Olympus, Center Valley, PA, USA). The positive area was calculated after binalizing the image using ImageJ [28]. The percentage of area of vascularization was averaged for each treatment group and a one-tailed student’s t-test was used to evaluate for statistical significance. 

## 4. Conclusions

Anti-VEGF therapy has become established as an anti-angiogenic cancer therapy [8,9]. Multiple drugs to block VEGF signaling have been developed and successfully used in cancer treatment, including bevacizumab and aflibercept [29,30,31,32,33]. One significant concern with these therapies is the development of resistance to anti-VEGFs during treatment [34]. Further evaluation of the VEGF signaling pathway has taken place to identify therapeutic alternatives to direct anti-VEGF therapy.

VEGFR2 plays an important role in cancer growth through two distinct mechanisms: a direct effect on certain tumor cells that express VEGFR2 and a pro-angiogenic effect on the vasculature supplying nutrients to the tumor [35,36,37]. Over the past decade, VEGFR-2 has been explored as a therapeutic target because of its role in angiogenesis. The primary modalities aimed toward this target have been anti-VEGFR2 antibodies, siRNAs, and small-molecule VEGFR2 inhibitors (e.g., sunitinib). These treatments have had success in a number of preclinical animal studies and clinical trials, both through direct and indirect modulation of tumor cells and vasculature [38,39,40,41,42]. On the other hand, some publications on anti-VEGF inhibitors have also reported either an increase in malignant features of the tumor or a mixed response to therapy [43,44,45,46]. While the reason for this is likely multifactorial, we suggest that the effect of the drugs on the balance of VEGFR2 membrane-bound and soluble isoforms may be partially contributory.

In a previous paper, we developed a morpholino that shifts expression of mVEGFR2 to sVEGFR2 by targeting alternative polyadenylation. sVEGFR2 is produced by utilization of polyadenylation signals within intron 13 in mice [18]. In our previous paper, we demonstrated that this latent polyadenylation site could be activated by blocking the upstream 5' splice site (exon 13-intron-13 junction) with a morpholino targeted to this sequence. Causing this alternative polyadenylation lead to decreased mVEGFR2 and increased sVEGFR2. We showed that this shift in gene expression led to decreased angiogenesis in a choroidal neovascularization model, corneal neovascularization model, and a corneal transplant model [18].

In the current paper, we show that the sVEGFR2-inducing morpholino decreases tumor vascularization and tumor growth in a mouse xenograft model. This gene therapy technique has the advantage of decreasing angiogenesis by both decreasing a pro-vascularization molecule (mVEGFR2) and increasing an anti-vascularization molecule (sVEGFR2). sVEGFR2 has been shown to bind to VEGF-A and VEGF-C [16,17]. The HCT116 cells used in this experiment do not express VEGFR2 in either isoform while mouse endothelial cells do. Therefore, is likely that the effects of the sVEGFR2-inducing morpholino are taking place in the mouse endothelial cells rather than the HCT116 cells. To further support this, the *in vitro* cell proliferation assays showed that the sVEGFR2-inducing morpholino does not affect HCT116 proliferation but does affect HAEC proliferation.

The existence of both membrane-bound and soluble isoforms of the same receptor is not unique to VEGFR2. Many other receptors have both of these isoforms, including VEGFR1 and Tie2 [47]. There are at least two distinct mechanisms for the creation of soluble and membrane-bound isoforms: Alternative splicing and shedding [47,48]. Shedding refers to post-translation cleavage of the extracellular portion of the receptor. Soluble Tie2 is an example of this [48]. Alternative splicing refers to pre-translational modification of mRNA to produce a soluble form, both VEGFR1 and VEGFR2 have been shown to have alternative splicing for soluble isoforms [18,49]. The role of shedding for production of sVEGFR2 has been discussed, but is not yet clear [50]. The morpholino presented in this paper targets alternative splicing and is not focused on shedding.

Future experiments are needed to further evaluate the utility of our morpholino in the treatment of cancer. Though in this paper we wanted to focus on the anti-angiogenic properties of our morpholino, xenograft experiments using cell lines that express mVEGRF2 on the tumor cells themselves may show an increased response to our treatment. Additionally, as lymphangiogenesis is important for metastasis, using the sVEGFR2-inducing morpholino in a metastatic model may show an additional benefit of this treatment by suppressing lymphangiogenesis, as sVEGFR2 has higher affinity for VEGF-C than VEGF-A [16]. Systemic injection of morpholino oligomers and other delivery techniques such as nanoparticles are also avenues of future research.
